# Down syndrome and congenital heart disease: perioperative planning and management

**DOI:** 10.1186/s40949-021-00061-3

**Published:** 2021-04-20

**Authors:** Dennis R. Delany, Stephanie S. Gaydos, Deborah A. Romeo, Heather T. Henderson, Kristi L. Fogg, Angela S. McKeta, Minoo N. Kavarana, John M. Costello

**Affiliations:** 1 Department of Pediatrics, Division of Pediatric Cardiology, Medical University of South Carolina, 10 McClennan Banks Drive, MSC915, Charleston, SC 29425 USA; 2 Department of Medicine, Division of Cardiology, Medical University of South Carolina, 30 Courtenay Drive, MSC 592, Charleston, SC 29425 USA; 3 Anesthesia and Perioperative Medicine, Division of Pediatric Anesthesia, Medical University of South Carolina, 10 McClennan Banks Drive, MSC940, Charleston, SC 29425 USA; 4 Department of Food and Nutrition, Sodexo, Medical University of South Carolina, 10 McClennan Banks Dr., MSC915, Charleston, SC 29425 USA; 5 Department of Surgery, Division of Pediatric Cardiothoracic Surgery, Medical University of South Carolina, 10 McClennan Banks Dr., MSC915, Charleston, SC 29425 USA

**Keywords:** Down syndrome, Trisomy 21, Congenital heart disease, Congenital heart surgery, Perioperative management

## Abstract

Approximately 50% of newborns with Down syndrome have congenital heart disease. Non-cardiac comorbidities may also be present. Many of the principles and strategies of perioperative evaluation and management for patients with congenital heart disease apply to those with Down syndrome. Nevertheless, careful planning for cardiac surgery is required, evaluating for both cardiac and noncardiac disease, with careful consideration of the risk for pulmonary hypertension. In this manuscript, for children with Down syndrome and hemodynamically significant congenital heart disease, we will summarize the epidemiology of heart defects that warrant intervention. We will review perioperative planning for this unique population, including anesthetic considerations, common postoperative issues, nutritional strategies, and discharge planning. Special considerations for single ventricle palliation and heart transplantation evaluation will also be discussed. Overall, the risk of mortality with cardiac surgery in pediatric patients with Down syndrome is no more than the general population, except for those with functional single ventricle heart defects. Underlying comorbidities may contribute to postoperative complications and increased length of stay. A strong understanding of cardiac and non-cardiac considerations in children with Down syndrome will help clinicians optimize perioperative care and long-term outcomes.

## Background

Down syndrome (DS) is the most common chromosomal abnormality with a prevalence of 11.8 per 10,000 live births [1]. Approximately 50% of newborns with DS have congenital heart disease (CHD) [2, 3]. The vast majority of these heart defects require surgical or transcatheter intervention, most commonly in infancy. Although many of the principles and strategies of perioperative evaluation and management for patients with CHD apply to those with DS, there are a number of specific issues that warrant special attention.

In this manuscript, for children with DS and hemodynamically significant CHD, we will summarize the epidemiology of heart defects that warrant intervention. We will review perioperative planning for this unique population, including anesthetic concerns, common postoperative issues, nutritional strategies, and discharge planning. Special considerations for single ventricle palliation and heart transplantation evaluation will also be discussed.

## Methods

The authors searched the published literature using a variety of medical subject terms to identify peer-reviewed articles on perioperative care issues relevant to the management of patients with DS and CHD, ranging in age from neonates to adults. We focused on original manuscripts published since 2010 but included a number of earlier relevant publications. Non-full text publications or languages other than English were excluded.

## History of congenital heart surgery in patients with Down syndrome

Although aggressive medical management and surgical intervention for CHD in patients with DS is routine practice in the current era, that was not always the case. John Langdon Down first described the features of DS in 1866 and Jerome Lejeune linked the syndrome to the chromosomal abnormality of trisomy 21 in 1959 [4]. Open heart surgery for most congenital heart defects became widely available in the 1960s and early 1970s. In the early years of congenital heart surgery, patients with DS were systematically not offered cardiac surgical intervention [5]. However, as outcomes for noncardiac conditions that afflict infants with DS improved and complete cardiac repair in infancy became more common for large septal defects, it was found that patients with DS generally tolerated surgery well. Societal attitudes changed, and increasingly more infants with DS and CHD were referred for surgery. Today, it is the standard of care to offer cardiac surgery to patients with DS with CHD that otherwise meet criteria for intervention.

## Epidemiology of CHD in patients with Down syndrome undergoing surgery

DS is present in nearly 10% of all children undergoing cardiac surgery, including approximately 2% of neonatal cases [6, 7]. Based upon statistics reported from the Society of Thoracic Surgeons Congenital Heart Surgery (STS-CHS) Database, the most common surgical procedures performed in children with DS are summarized in Table 1 [6].

**Table 1:** Most common cardiac surgeries in patients with Down syndrome as summarized in the STS-CHS Database [6]

Procedure	% of patients with Down syndrome
Complete atrioventricular septal defect repair	33
Ventricular septal defect closure	19
Mitral valve repair/replacement	7
Partial atrioventricular septal defect repair	6
Patent ductus arteriosus ligation	4
Tetralogy of Fallot repair	4
Atrial septal defect closure	4
Coarctation/arch repair	2
Tricuspid valve repair/replacement	2
Tetralogy of Fallot- atrioventricular septal defect repair	1

*STS-CHS* Society of Thoracic Surgeons Congenital Heart Surgery

## Special considerations for perioperative planning

Preoperative evaluation and planning for any patient being considered for a cardiac surgical procedure must be thorough and consider the patient’s medical and surgical history, cardiac anatomy, and non-cardiac comorbidities. Additional details regarding cardiovascular disease and extracardiac comorbidities warrant specific attention when planning surgery for patients with DS and CHD.

### Simple left to right shunts

Ventricular septal defect (VSD), atrial septal defect (ASD), and patent ductus arteriosus (PDA) are common types of CHD present in children with DS [6, 8, 9]. In patients with ASD and VSD, data from the STS-CHS Database indicate that those with DS were younger and weighed less for age at time of surgery compared with patients without genetic anomalies. Specifically, average ages at time of repair of ASD was 1.2 years versus 4.1 years, and average ages at time of VSD repair was 4.8 months versus 7.4 months (DS versus non-DS respectively) [6].

### Atrioventricular septal defects

Due to the increased frequency of endocardial cushion defects with DS, evaluation of left to right shunts should specifically evaluate for inlet-type VSDs, primum-type ASDs, and partial, transitional, or complete atrioventricular septal defects (AVSD). With these defects, special attention should be given to assess for conoseptal malalignment in VSDs, additional muscular VSDs, and crossing atrioventricular valve chords in inlet-type VSDs.

Repair of complete AVSD is the most common cardiac procedure for patients with DS [6]. As with any preoperative cardiac work-up of complete AVSD, detailed evaluation should be completed of the common atrioventricular valve (AVV), ventricular balance, and ventricular outflow tracts. Structurally, patients with DS have lower rates of left AVV dysplasia (3% with DS vs. 24% with non-DS), unbalanced ventricles (3% with DS vs. 25% with non-DS), and left-sided obstructive lesions (1% with DS vs. 30% with non-DS) when compared to those with complete AVSD without genetic anomalies [10–12]. Additionally, the AVVs in patients with DS are more likely to be Rastelli Type A, with a divided anterior bridging leaflet with connection to the crest of the ventricular septum [13]. Excluding a patent foramen ovale or a small additional secundum ASD, nearly half of patients with DS undergoing AVSD repair have one or more additional cardiac procedures performed intraoperatively, including PDA ligation, repair of aortic arch anomaly, closure of additional VSD, baffle of anomalous systemic venous drainage, repair of pulmonary venous stenosis, and subaortic stenosis resection [14].

Compared to patients without chromosomal abnormality, those with DS tend to present for AVSD repair surgery at the same age but lower average weight [6]. Growth failure at surgery is high at 71% for infants with DS versus 21% of those without chromosomal abnormality [14]. Of note, patients with DS tend to have significant catch up growth after surgery but a large proportion still have growth failure (27% in DS vs. 7% in non-DS) [14].

### Tetralogy of Fallot

In addition to endocardial cushion defects, the presence of conoseptal malalignment must also be evaluated. TOF or coexistent TOF and AVSD are more common in children with DS than in children without genetic abnormalities [6]. Overall, those with TOF are repaired around the same age but have a lower weight for age [6].

### Single ventricle palliation

**Fig. 1:**
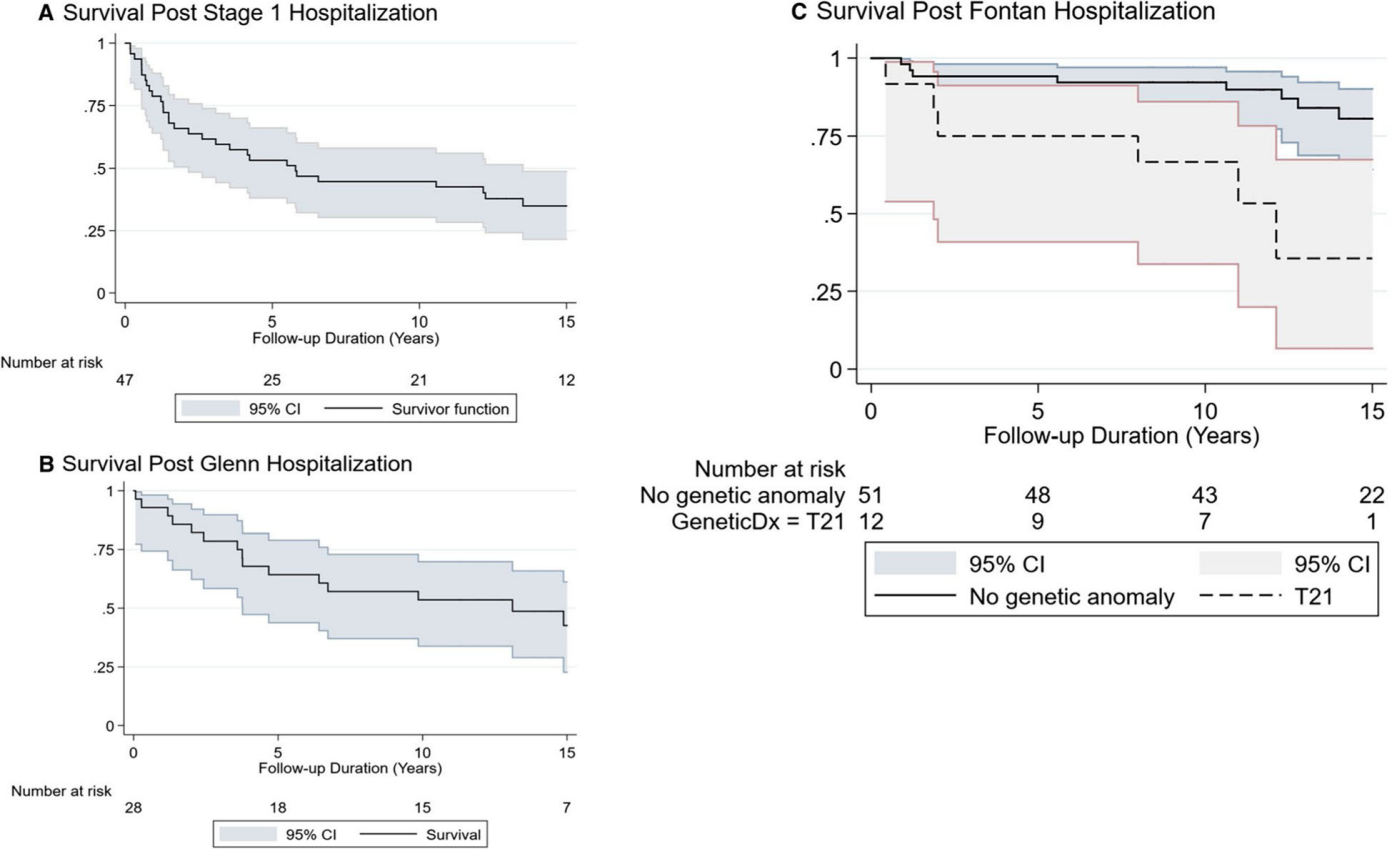
Kaplan-Meier transplant-free survival plot conditioned on hospital discharge: **a** after the Stage 1 procedure in patients with DS with functional single ventricle heart defect, **b** after Glenn procedure in patients with DS with functional single ventricle heart defect, and **c** after Fontan procedure in patients with DS with functional single ventricle heart defect. DS, Down syndrome; CI, Confidence interval [15]. (Used with permission)

Single ventricle lesions are occasionally seen in patients with DS. Structurally, patients with DS are more likely to have left ventricle dominant lesions, while patients without DS are more likely to have right ventricle dominant or complex single ventricle lesions, including transposition of the great arteries or left-sided obstructive lesions [15, 16]. Despite generally having more favorable anatomy for single ventricle palliation, the reported outcomes in the literature indicate that mortality is relatively high compared to patients without DS. A STS-CHS Database study found that there was increased operative mortality in patients with DS at every stage of single ventricle palliation (Stage 1 palliation: 73% in DS vs. 19% in non-DS, Glenn operation: 19% in DS vs. 2% in non-DS, and Fontan operation: 24% in DS vs. 2% in non-DS) [6]. Using Pediatric Cardiac Care Consortium (PCCC) data, Petersen et al. showed that patients with DS and functional single ventricle heart defects had high in-hospital first-stage mortality at 24%, and for those undergoing a Norwood procedure, specifically, hospital mortality was 68% [15]. These investigators also found that only 21% of patients included in the initial cohort completed the Fontan procedure, and of those who did not go to Fontan, 50% were ineligible due to hemodynamic or anatomic concerns [15]. The Kaplan-Meier Survival following each stage of single ventricle palliation for DS patients is depicted in Fig. 1.

Risk stratification in patients with DS and functional single ventricle heart defects can be challenging due to small sample sizes in most studies, but pulmonary hypertension (PH) is likely one of the main contributing factors to mortality [6, 16, 17]. Colquitt et al. showed that patients with DS and single ventricle anatomy have minimal mortality beyond 2 years of age if their pulmonary vascular resistance (PVR) is less than 3 indexed Woods units in their first year of life [18]. In fact, when adjusting for PVR, this study found that DS is not associated with increased mortality compared to other non-syndromic patients undergoing the single ventricle palliation at their center [18]. These data suggest that for patients with DS and functional single ventricle heart defects, careful patient selection is critical to achieving acceptable outcomes. One must consider the potential for decreased responsiveness to PH therapies found in patients with DS (described below) when planning on staged procedures [19–21]. In children with DS, performing a pulsatile Glenn operation or fenestrated Fontan operation, if anatomy and hemodynamics allow, has been shown to decrease perioperative and late mortality [22]. If hemodynamics are borderline after Glenn procedure, one should strongly weigh the risks versus benefits of proceeding to Fontan, as one report suggested that Fontan completion was not associated with improved survival compared with sustained Glenn physiology [15].

### Pulmonary hypertension

In patients with DS who are being evaluated prior to a cardiac operation, the work-up should closely evaluate for signs of PH. More specifically, the concern should be for pulmonary vascular disease (PVD), meaning elevated pulmonary arterial pressures in the context of changes in the pulmonary vascular bed leading to increased PVR. Children with DS are at more than 20-fold greater risk for having PH when compared to the general population, and those with CHD are at even greater risk [23]. Lung disorders including relative underdevelopment of the pulmonary vascular bed (pulmonary hypoplasia) and chronic issues with upper airway obstruction are likely contributory (World Health Organization Group 3 PH). Left to right intracardiac shunts with longstanding overcirculation create increased pulmonary artery pressure, shear stress, endothelial dysfunction, vascular remodeling, and altered vasoactive mediator expression (World Health Organization Group 1 PH). Additionally, patients with obstructed pulmonary venous return, mitral stenosis, or left ventricular outflow obstruction (critical aortic valve stenosis or coarctation of the aorta) may develop increased PVR (World Health Organization Group 2 PH).

Studies from the early 1990s have shown higher PVR that is more likely to be irreversible in DS patients, and this phenomenon may develop as early as 6 months of age [24, 25]. These observations have been correlated more recently in a STS-CHS Database report which found higher rates of PH present in patients with ASDs or VSDs and DS compared to those without genetic anomalies [6]. Surprisingly, higher rates of PH were not found in patients with AVSD and DS, likely related to the fact that, in the current era, most undergo repair within the first 6 months of life [6]. In fact, early definitive repair (i.e., within the first 6 months of life) can reduce the incidence of postoperative PH [26]. A combination of anecdotal experience and data from early case series has prompted most centers into performing complete AVSD and large VSD repairs at 4–6 months of age.

Clinically, suspicion for PH should be increased in children with anatomically large left to right shunts if there is a lack of signs and symptoms of pulmonary overcirculation, lack of failure to thrive, or if increasing cyanosis is present. By echocardiogram, evidence of PH can be shown using a variety of methods depending on the type of lesion. In the presence of a restrictive VSD or intact ventricular septum without right ventricular outflow tract obstruction (RVOTO), signs of PH include a tricuspid regurgitation jet estimating right ventricular pressure greater than half systemic or by intraventricular septal flattening. If there is a nonrestrictive VSD or large PDA, PH may be suggested by right to left shunting at ventricular or great vessel level (in the absence of RVOTO) or an elevated early diastolic pulmonary regurgitation gradient. Finally, in the absence of left sided obstructive lesions, right to left shunting at the level of the PDA may also provide evidence of PH. As shown by the number of caveats based on type of CHD noted above, these echocardiographic findings only suggest that PVR is elevated. If there is clinical or echocardiographic evidence of PH or the patient is older with a longstanding left to right shunt lesion, then cardiac catheterization should be considered to quantify PVR, and, if it is elevated, pulmonary vaso-reactivity testing with oxygen and nitric oxide is typically performed. Alternatively, given the risks associated with cardiac catheterization, an infant with initial predominant right to left shunting can be placed on oxygen while in clinic, observed to see if the oxygen saturation increases, and the direction of the shunt on echo can be reassessed. If the direction of the shunt reverses becoming left to right, a cardiac catheterization can likely be avoided. Of note, there is some evidence that patients with DS may have less response to pulmonary vasodilators, including nitric oxide [20]. In the STARTS trial, sildenafil was tolerated well in patients with DS, but led to little improvement of PVR [19]. Similarly, in a study by Duffles et al., bosentan did not have an effect on exercise capacity or quality of life in adults with pulmonary arterial hypertension related to CHD and DS [21].

Following cardiopulmonary bypass (CPB), PH may be caused by a combination of preoperative, intraoperative, and postoperative factors. DS is a known risk factor for severe postoperative PH [27]. Yet, even in the absence of DS, CPB is associated with increased PVR in infants and children [28–30]. The presence of significant PH soon after weaning from CPB is predictive of subsequent PH in the ICU and the need for prolonged ventilatory support. Residual lesions, including large left to right shunts, obstruction to pulmonary venous or distal pulmonary arterial blood flow, or left-sided obstructive lesions may cause PH. Noxious stimuli, particularly suctioning of the endotracheal tube, may trigger a pulmonary hypertensive crisis. PH may manifest as low cardiac output following a biventricular repair, particularly when both septa are completely intact, or as excessive cyanosis in patients with palliated single ventricle physiology. In general, children with PH are at increased risk of perioperative complications [31, 32]. A combination of relatively simple postoperative strategies should be sufficient both to prevent and to treat pulmonary hypertensive crises in many at-risk patients (Table 2) [33].

**Table 2:** Critical care strategies for treatment of PH [33]

Encourage	Avoid
1. Anatomic investigation	1. Residual anatomic disease
2. Right to left atrial pop off	2. Intact atrial septum
3. Sedation/analgesia	3. Agitation/pain
4. Moderate hyperventilation	4. Respiratory acidosis
5. Moderate alkalosis	5. Metabolic acidosis
6. Adequate inspired oxygen	6. Alveolar hypoxia
7. Normal lung volumes	7. Atelectasis or overdistension
8. Optimal hematocrit	8. Excessive hematocrit
9. Inotropic support	9. Low output and coronary perfusion
10. Pulmonary vasodilators	10. Pulmonary vasoconstrictors

### Non-cardiac comorbidities

Features of DS may manifest in nearly every organ system, and thus a comprehensive assessment is required prior to taking a patient with DS to the OR [34]. Specifically, one should consider the potential for problems within the gastrointestinal, respiratory, immunologic, hematologic, endocrine, and neurologic systems. Generally, clinicians involved in preoperative evaluation should refer to the American Academy of Pediatrics guidelines for “Health supervision for children with Down syndrome” for age-appropriate screening [35].

#### Gastrointestinal

After CHD, abnormalities of the gastrointestinal tract are the most common anomaly associated with DS, being present in 6% of cases in one large European registry study [9]. These include duodenal atresia, duodenal webbing, annular pancreas, anal atresia, Hirschsprung disease, and tracheoesophageal fistula/esophageal atresia. If there are feeding concerns preoperatively or the neonate fails to pass meconium, there should be a low threshold to further evaluate for these anomalies.

#### Respiratory

The respiratory manifestations of DS contribute significantly to morbidity and reduced survival in this population [36–38]. The anatomic features are discussed below in the section on anesthesia management. Functionally, obstructive sleep apnea (OSA) has a high prevalence of about 76–97% in children with DS who snored and 55% in those without snoring [39, 40]. The risk of OSA increases with BMI and age [41], although OSA may be more severe if it presents earlier [40]. If OSA is found preoperatively, consideration for adenotonsillectomy should be taken, although the timing of that procedure relative to an upcoming cardiac operation will require clinical judgment. Children with DS and OSA who underwent adenotonsillectomy showed significant improvement of obstructive and central apneic indices [42–45]. Additionally, OSA likely plays a long-term role in the development of PH, and adenotonsillectomy may help halt or reverse the progression of PH and right heart disease [46, 47].

#### Immunologic/hematologic

Children with DS are also more likely to develop infections and to take longer to recover from them [48, 49]. Multiple factors are contributory, as children with DS may have abnormalities of the immune system with T and B cell lymphopenia, secondary immunodeficiency due to nutritional and metabolic factors, and anatomic abnormalities with small ear canals, tracheomalacia, tracheal bronchus, and gastroesophageal reflux [48]. Overall, the most frequent type of infections involve the respiratory tract, especially the upper respiratory tract [49, 50]. Additionally, patients with DS and acute respiratory tract infection are more likely to develop acute lung injury or acute respiratory distress syndrome [51]. Careful history for recent infections or history of infections requiring hospitalization should be obtained preoperatively.

Neonates with DS can have neutrophilia, thrombocytopenia/thrombocytosis, polycythemia transient myeloproliferative disorder, and congenital leukemia [52]. Children with DS have a 20-fold increased risk of leukemia, with acute myeloblastic leukemia as the most common type, and the highest incidence occurs in children younger than 5 years [53]. Interestingly, although the rates of blood cancers are higher in children with DS, most other cancers tend to be lower than non-DS population [53, 54]. For these reasons, a complete blood count should be obtained before surgery, and abnormal blood counts should be discussed with hematologist.

#### Endocrine

Thyroid disorders are common in DS. Hypothyroidism has a prevalence of 4–18% [55, 56]. If not assessed on the state newborn metabolic screen, TSH should be obtained in the neonatal period. The risk for hypothyroidism increases with age, and TSH should be followed annually [35, 57]. Dry skin, thin hair, poor appetite, constipation, poor energy, hypotonia, bradycardia, and behavior changes may be signs of hypothyroidism, but are not always present [58]. Thyroid hormones are important for cardiovascular effects of decreasing afterload and increasing heart rate, preload, and contractility. If there are any signs or symptoms of hypothyroidism or a level has not been recently checked, consider checking a TSH with preoperative labs.

Though not generally a problem in the operative room, hypothyroidism can contribute to hypotension post-operatively and is easily treated with triiodothyronine therapy when identified. Cardiopulmonary bypass has been shown to decrease thyroid hormone levels [59]. A triiodothyronine level less than 0.6 has been associated with longer mechanical ventilation and ICU LOS, with higher vasoactive drip and furosemide cumulative doses [59]. A separate study however, showed no significant cardiac impairment in patients with DS and subclinical hypothyroid [60]. Additionally, replacement of triiodothyronine post-bypass has shown little benefit [61]. Overall, the literature does not support routine use of intraoperative or early postoperative triiodothyronine supplementation, but larger studies are needed.

#### Neurologic

The degree of cognitive impairment can range from mild (intelligence quotient 50–70) to severe (intelligence quotient 20–35) [35]. Clinicians should also be aware of the 1–13% prevalence of seizures and frequent hearing and vision impairment with a prevalence of 60–75% [35]. Preoperatively, it is useful to understand the child’s cognitive function, likes and dislikes, and any physical handicaps, and to deliver this information to the anesthesia and postoperative care teams.

### Adults with Down syndrome and congenital heart disease

Special attention should be allocated to the surgical and anesthetic preoperative assessment for adult patients with DS undergoing congenital cardiac surgery. As described above, comorbidities such as obstructive sleep apnea, hypothyroidism, and epilepsy occur with high frequency and may significantly impact a patient’s perioperative course [62]. Hypothyroidism can develop slowly over time, and can be difficult to detect clinically in adults with DS given overlapping symptomatology with other aging processes [63, 64].

Despite possessing increased rates of many traditional risk factors for acquired cardiovascular disease such as obesity, diabetes, sedentary lifestyle, and evidence of early aging within other organ systems, atherosclerosis is surprisingly uncommon in adults with DS [65–68]. In contrast to the general population of adults with similar risk profiles undergoing cardiac surgery, there is no recommendation for specific preoperative screening for coronary artery disease in adults with DS. As mentioned, diabetes does occur more commonly in patients with DS and would require additional perioperative monitoring and treatment to maintain goal glucose levels [69]. The team should note patients’ fasting serum glucose levels on preoperative labs and, if elevated, consider additional lab screening with a hemoglobin A1C.

There should be a high suspicion for dementia in patients with DS over 40 years of age. Alzheimer’s disease occurs in nearly 25% of adults with DS ages 40 to 49 years and in up to 55% of those ages 50 to 59 years [70]. Preexisting dementia is a significant risk factor for delirium in subacute care settings, and delirium confers broadly worse clinical and functional patient outcomes [71]. Identification of baseline dementia is additionally relevant when considering expected patient behavior and communication strategies postoperatively. Similarly, preoperative inquiry of visual and hearing abilities is important given prevalence of sensorineural hearing loss and early cataract formation in adults with DS [62]. Lastly, there should be close inspection of dental health on a preoperative assessment given the higher frequency of gingivitis and periodontal disease in adults with DS [72]. Such dental issues may raise a patient’s risk of spontaneous bacterial endocarditis postoperatively and, if possible, should be treated prior to certain cardiothoracic interventions.

### Cardiac transplantation in children with Down syndrome

Sometimes surgical repair or palliation is not a good option for patients with DS, and heart transplant may be considered. There is wide variation among transplant centers of how intellectual and developmental disabilities are factored into transplant evaluations and determination of candidacy. However, denying a patient with DS that would otherwise benefit from transplant the possibility of the same evaluation as a non-DS patient is unjust and prohibited by Americans with Disabilities Act (ADA) [73]. In 1995, Sandra Jensen, a 34 year-old with DS and CHD was denied heart-lung transplant solely based on her underlying chromosomal abnormality. After a national campaign protesting this discrimination, she underwent successful transplant in 1996, becoming the first patient in the world with DS to receive a life-saving organ transplant [73, 74].

Since this time, several small studies report patients with DS receiving successful solid organ transplants with equivalent outcomes to non-DS patients [73, 75–78]. Intellectual disability alone does not affect short- and medium-term outcome in children after heart transplant, supporting the concept that intellectual delay that occurs in DS should not alone impact eligibility for transplant [78–81]. Overall, little is known specifically about heart transplant outcomes in patients with DS. A study by Broda, et al. evaluated cardiac transplant outcomes in children with various chromosomal abnormalities and found only 64 of 3080 (2%) pediatric heart transplants were performed in children with various chromosomal abnormalities, and only five of these patients had DS, one of whom died prior to hospital discharge. Given the small numbers it was hard to make conclusions about prognosis [76].

Although intellectual disability should not preclude patients with DS from a thorough transplant evaluation and possible listing, many other factors need to be considered. Given the scarcity of donor organs, potential transplant recipients undergo a rigorous evaluation to determine their candidacy for transplantation. The evaluation goals are to determine whether a patient that meets medical criteria for transplant would benefit from the transplant surgical procedure and would also tolerate post-transplant medical therapies to extend both the quantity and quality of life. The evaluation must consider medical co-morbidities and behavioral and psychosocial factors.

All patients undergo evaluation of their other organ systems as part of the transplant evaluation process. Of particular concern is underlying pulmonary and renal dysfunction, as well as the strength of the immune system given increased risk of infection, hematologic abnormalities and malignancy post-transplant [9, 81–83]. Significant PVD is a relative contraindication for heart transplant. It is well known that DS is associated with an increased risk of malignancy, especially leukemia/lymphoma, some of which may ultimately be treated with bone marrow transplantation. These patients have worse outcomes compared to their non-DS leukemia patients [84]. Other hematologic and immunologic abnormalities have been reported in DS including a higher prevalence of anemia, including aplastic anemia, as well as underlying immune dysfunction increasing the risk of infections, duration of infections and complication rate from various infections [48, 85–88]. Although these comorbidities are significant, they are not absolute contraindications to heart transplant. However, the risks must be considered, especially when multiple organ-systems are involved. These diseases are equally important in the post-transplant period and may require avoidance of induction therapy and alteration of maintenance immune-suppressive therapy long term. Increased screening for infection, hematologic abnormalities and for malignancy should be performed.

In addition to medical co-morbidities, DS patients’ families and support systems are a crucial part in the evaluation process. Education of families about the individual patient’s risks and potential benefits of transplantation is an essential part of the evaluation process so they can make the best decision for their family. There are some families that may decide they are not equipped to carry the life-long burden of post-transplant care and should be supported in making that decision. However, if the family is interested, patients with DS and end-stage heart failure refractory to medical and other surgical therapies should be evaluated for heart transplant, regardless of underlying Trisomy 21 and intellectual disability. Patients with DS should not be denied access to a scarce resource based on their syndrome or disability, despite earlier practice and underlying bias about providers’ perceived quality of life in DS patients [73, 89, 90].

## Intraoperative management and anesthesia

As is evident from the proceeding paragraphs, the implications of DS when considering an anesthetic are vast and require additional assessment and management [91].

### Upper airway obstruction

Patients with DS have a high incidence of upper airway obstruction. Characteristic anatomic features include flattened nasal bridge, mid face and mandibular hypoplasia, macroglossia, soft palate hyperplasia, tracheal bronchus, tracheal and subglottic stenosis, as well as laryngomalacia and tracheomalacia [92, 93]. Further contributing to upper airway obstruction are tonsillar-adenoidal hypertrophy, pharyngeal muscle hypotonia, and obesity affecting the soft tissues of the airway. These features together can lend to upper airway obstruction or complete collapse upon induction of general anesthesia [94]. For this reason the anesthesia provider must be prepared with oral and nasal airways to help facilitate bag mask ventilation, readily use positive pressure ventilation to help stent lax tissues open and be prepared for a challenging laryngoscopy by having additional airway equipment available. Upon emergence from general anesthesia, patients with DS and pre-existing upper airway obstruction may continue to have upper airway obstruction which can be exacerbated by sedatives and analgesics, and thus the need for post-operative ventilatory support will need to be evaluated in these situations [31, 92]. When considering subglottic stenosis an appropriately sized endotracheal tube needs to be selected and a cuff leak auscultated at 20 mmHg [95]. An inappropriately oversized endotracheal tube can lead to post-extubation stridor.

### Cervical spine instability

There is a risk of atlantoaxial and atlanto-occipital instability in patients with DS. The rate of occurrence varies widely in the literature. Identification of patients with instability is a challenge, as very few of these patients will show symptoms and obtaining adequate imaging is difficult. Previously the American Academy of Pediatrics recommended lateral cervical spine X-rays for children with DS between 3 and 5 years of age, but recent guidelines no longer support this recommendation for asymptomatic children [35]. In the absence of clear guidelines, some providers recommend performing a thorough neurologic exam in patients with DS presenting for general anesthesia that will require instrumentation of the airway [91]. If an abnormality in exam is identified, the need for further imaging should be discussed between the anesthesia provider and the surgeon. When instrumenting the airway, consideration should be made to maintain in-line neck stabilization or consider a fiberoptic intubation.

### Hemodynamic changes with anesthesia

Independent of structural cardiac disease, patients with DS experience hemodynamic changes upon inhalation induction of general anesthesia. They experience a higher incidence of bradycardia with a sevoflurane inhalation induction than do patients without DS [31, 96, 97]. For this reason some practitioners will pre-treat with atropine or glycopyrrolate. Some patients with DS can present a challenge with vascular access and as such an intramuscular dose of atropine may be required on induction of anesthesia [96].

### Gastrointestinal

Patients with DS have a higher rate of duodenal obstruction. These patients often present to the operating room dehydrated with electrolyte abnormalities. They frequently require a rapid sequence induction to secure their airway and avoid aspiration. Intraoperative resuscitation and management of electrolytes is often necessary [31].

### Cardiopulmonary bypass

Little data exists on differences on cardiopulmonary bypass between patients with and without DS. STS-CHS data shows slightly longer cardiopulmonary bypass and cross-clamp time for VSD closures in patients with DS, slightly shorter times for ASD repairs, but no significant difference in AVSD or TOF repairs [6].

## Postoperative care

### Morbidity and mortality

Since children with DS started undergoing cardiac surgery, many studies have compared their operative outcomes. Overall, most studies have found that operative mortality for patients with DS is the same or better than their peers with normal chromosomes [6, 12, 98–101]. Of note, the majority of these studies focused on patients undergoing biventricular repairs. As discussed earlier, patients with functional single ventricle heart defects and DS have decreased long-term survival. Despite good mortality outcomes in biventricular repairs, patients with DS may have increased postoperative complications and length of stay [6, 98, 99, 102]. Postoperative complications may include infection, respiratory complications, reintubation, prolonged ventilation, PH, renal insufficiency, and chylothorax. Many of these complications can be attributed to underlying comorbidities, as discussed earlier. For example, the increased rate of chylothorax is likely related to abnormalities of the lymphatic system more common in children with DS [103]. Despite a propensity to develop this postoperative complication, patients with chylothorax and DS generally have the same mortality, time in ICU, and duration of hospital stay as those patients with chylothorax and without DS [101].

### Down syndrome-specific post-operative cardiac complications

The importance of chromosome 21 with embryonic heart development is evident in the prevalence of CHDs in individuals with DS. This fact lends to further question syndromic implications in cardiac-specific postoperative outcomes. Studies surmise that reoperation on residual cardiac lesions is no more likely in patients with DS compared to normal karyotype patients. Furthermore, there are some compelling claims that patients with DS in fact have better freedom from reoperation. Despite being typically younger and of lower weight than non-DS patients at time of surgery [6], patients with DS have demonstrated lower rates of reoperation after all types of cardiac surgical repair compared to non-syndromic patients [12]. This was especially true for mitral valve surgery, prosthetic replacement, and recurrent subaortic stenosis. Other assessments specific to post-operative outcomes following AVSD repairs show conflicting results. Some demonstrate no differences in post-operative left atrioventricular valve dysfunction between patients with DS and those without DS [104, 105], while others showed significantly less need for valve reoperation in patients with DS [10, 106]. Overall, the available data indicate that the DS population is not expected to have higher rates of reoperation following CHD repair compared to non-syndromic patients.

One interesting caveat among post-operative outcomes in patients with DS is the risk of damage to the cardiac conduction system. Postoperative heart block resulting in permanent pacemaker placement seems to occur more commonly in DS patients undergoing VSD closure than normal karyotype patients, with DS being independently predictive of this outcome when accounting for patient age or weight at surgery [6, 107]. The reason behind this finding remains unclear as there are no known anatomic abnormalities of the conduction system in patients with DS with perimembranous VSD. One possible explanation is that the perimembranous VSD may have some inlet extension and therefore displace the atrioventricular node inferiorly. The repair should be approached with the same precautions one would take while closing the ventricular septum component of a complete AVSD. There is atypical development of the atrioventricular node and ventricular conduction system in patients with DS with AVSD [108]. Studies have not consistently shown increased prevalence of complete heart block or other significant bradyarrhythmia in patients with DS after AVSD repair versus non-syndromic patients, including early and long-term follow-up [109–111].

### ECMO

Extracorporeal membrane oxygenation (ECMO) may be used to provide circulatory support for children with DS who are recovering from cardiac surgery. Data from the Extracorporeal Life Support Organization (ELSO) Registry indicate that the use of ECMO for cardiac support in children with DS has increased in recent years [112]. Of 300 children with DS whose data were entered into the ELSO Registry after receiving ECMO support for a cardiac indication between 1983 and 2013, 136 (39%) survived to hospital discharge [112]. The most common underlying cardiac diagnoses in these patients were partial or complete AVSD (38%) and TOF (13%) [112]. In another analysis of ELSO Registry data from 1998 through 2011, Gupta et al. compared children with DS who underwent cardiac surgery and were supported with ECMO (*n* = 121) with the children supported by ECMO post-cardiac surgery who did not have DS (*n* = 2694). They found that the patients with DS were older than the control group (median 133 vs 41 days), and that mortality was lower in the DS group (44%) compared with the control group (55%; *p* = 0.01) [113]. These survival rates are similar though to those reported for all patients with CHD in the ELSO registry, for whom survival to hospital discharge is 54% [114].

### Pain control

Pain control is an important component of postoperative management of children with DS. These patients often have a reputation for being difficult to sedate or requiring large amounts of pain medications. Multiple studies have evaluated this belief, showing some evidence that alteration in the cerebral cortex concentration in opioids and opioid receptors may exist in children with DS [115]. Gakhal, et al. found that the average rate of continuous morphine infusion after cardiac surgery was higher in the DS group, although this was not statistically or clinically significant until the third postoperative day, when patients with DS were more likely to still be receiving morphine [116]. They also showed the patients with DS were more likely to receive additional sedatives and skeletal muscle relaxants [116]. On the contrary, other studies evaluating cardiac and non-cardiac surgeries have shown similar overall opioid usage in patients with DS to those without DS [117, 118]. Additionally, the pharmacokinetics and pharmacodynamics of opioids in patients with DS versus those without DS do not significantly differ [119, 120]. Similarly, alternatives to opioids, such as acetaminophen, have similar pharmacokinetics in patients with and without DS [121]. Finally, dexmedetomidine is another sedative medication that is both safe and efficacious in patients with and without DS, although younger patients with DS may be more likely to experience significant bradycardia [122].

### Nutrition considerations

In the neonatal period, all patients with DS have higher incidence of low birth weight, hypotonia, impaired oral motor function, hypothyroidism, and gastrointestinal abnormalities that can contribute to poor growth [123–125]. Weight for age at time of cardiac surgery is often lower for patients with DS for all procedures, but this issue may also be an indication for earlier surgical referral [6]. Though impaired weight and height of children with DS and CHD are common, they have been shown to recover to normal values within 6 months post operatively [126].

Placement of gastrostomy tubes before or after cardiac repair is often necessary for the optimization of nutrition and growth in patients with DS, particularly in patients with AVSD and TOF [127, 128]. However, gastrostomy tube placement rate in patients with DS and CHD is similar to rate in DS patients without CHD [124]. When estimating energy requirements in infants with DS, resting energy expenditure (REE) without surgical repair is slightly lower due to decreased muscle tone [129]. This finding has not been replicated in CHD literature, as patients with chromosomal abnormalities are often excluded from these studies. Close attention should be paid to post-operative anthropometrics to avoid overfeeding in this population.

In adolescence and adulthood, patients with DS have a higher prevalence of overweight and obesity. Malnutrition followed by overweight and obesity may enhance risk to cardiovascular health compared to maintaining the same nutrition status over a period of time [130]. Use of DS specific growth charts are important for nutrition screening to accurately assess for both undernutrition and overweight/obesity and therefore to design a more targeted intervention [125]. In the post-operative adolescent or adult with DS, managing acute needs and transitioning to a general well-balanced diet with regular physical activity is important for prevention of longer-term cardiovascular disease [131].

### Discharge planning

Several considerations should be made when preparing patients with DS for hospital discharge following cardiac surgery. As previously stated, patients with DS are more susceptible to bacterial and viral infections, attributed to a disordered immune system [132]. Additionally, viral respiratory infections may have profound effects on patients with CHD [133]. Patients with DS are particularly at high risk of severe respiratory syncytial virus (RSV) infection and worse outcomes when compared to patients without DS [134]. Careful and thorough education should be provided to parents regarding preventative measures to reduce exposure to seasonal viruses, the importance of adhering to the recommended immunization schedule, and, if applicable, inclusion criteria for Palivizumab, a humanized monoclonal antibody used for prophylaxis against RSV [134].

For neonates who have never been discharged to home, discharge planning should include referrals to developmental clinics specializing in DS care and therapeutic services such as occupational, physical and speech therapies. Available resources including local chapters affiliated with the National Down Syndrome Society and other local support groups should be provided to parents [135].

## Conclusions

DS is a common chromosomal anomaly frequently associated with CHD. It also has broad effects on nearly all other body systems. Careful planning for cardiac surgery is required, evaluating for all cardiac and noncardiac disease, with careful consideration of the risk for PVD. Overall, the risk of mortality with cardiac surgery is no more than the general population, except for those with functional single ventricle heart defects. Underlying comorbidities may have a role in increased postoperative complications and increased length of stay. Communication with family to learn the patient’s history as well as to discuss risks is essential. Cardiac surgery and perioperative care of the patient with DS and CHD has progressed significantly in the past 50 years. Future advancements will likely involve improved PH management, greater understanding of heart transplant in patients with DS, development of better medical and surgical management of single ventricles, and enhanced coordination of care for these complex patients.

## Data Availability

Not applicable.
